# Extended robot‐assisted laparoscopic prostatectomy and extended pelvic lymph node dissection as a monotherapy in patients with very high‐risk prostate cancer Patients

**DOI:** 10.1002/cam4.4308

**Published:** 2021-09-25

**Authors:** Noriyoshi Miura, Naoya Sugihara, Keisuke Funaki, Toshio Kakuda, Kanae Koyama, Ryuta Watanabe, Yuichiro Sawada, Terutaka Noda, Kenichi Nishimura, Tetsuya Fukumoto, Yuki Miyauchi, Tadahiko Kikugawa, Takashi Saika

**Affiliations:** ^1^ Department of Urology Ehime University Graduate School of Medicine Toon Ehime Japan

**Keywords:** extended pelvic lymph node dissection, extended robot‐assisted laparoscopic prostatectomy, locally advance, prostate cancer, very‐high risk

## Abstract

**Background:**

Patients with very‐high‐risk prostate cancer (VHRPCa) have earlier biochemical recurrences (BCRs) and higher mortality rates. It remains unknown whether extended robot‐assisted laparoscopic prostatectomy (eRALP) without neoadjuvant or adjuvant therapy can improve the outcomes of VHRPCa patients. We aimed to determine the feasibility and efficacy of eRALP as a form of monotherapy for VHRPCa.

**Methods:**

Data from 76 men who were treated with eRALP without neoadjuvant/adjuvant therapy were analyzed. eRALP was performed using an extrafascial approach. Extended pelvic lymph node (LN) dissection (ePLND) included nodes above the external iliac axis, in the obturator fossa, and around the internal iliac artery up to the ureter. The outcome measures were BCR, treatment failure (defined as when the prostate‐specific antigen level did not decrease to <0.1 ng/ml postoperatively), and urinary continence (UC). Kaplan–Meier, logistic regression, and Cox proportional‐hazards model were used to analyze the data.

**Results:**

The median operative time was 246 min, and median blood loss was 50 ml. Twenty‐one patients experienced postoperative complications. Median follow‐up was 25.2 months; 19.7% of patients had treatment failure. Three‐year, BCR‐free survival rate was 62.0%. Castration‐resistant prostate cancer‐free survival rate was 86.1%. Overall survival was 100%. In 55 patients who had complete postoperative UC data, 47 patients (85.5%) recovered from their UC within 12 months. Clinical stage cT3b was an independent preoperative treatment failure predictor (*p *= 0.035), and node positivity was an independent BCR predictor (*p *= 0.037). The small sample size and retrospective nature limited the study.

**Conclusions:**

This approach was safe and produced acceptable UC‐recovery rates. Preoperative seminal vesicle invasion is associated with treatment failure, and pathological LN metastases are associated with BCR. Therefore, our results may help informed decisions about neoadjuvant or adjuvant therapies in VHRPCa cases.

**Precis:**

Extended robot‐assisted laparoscopic prostatectomy and extended pelvic lymph node dissection without adjuvant therapy is safe and effective for some patients with very‐high‐risk prostate cancer. The clinical stage and node positivity status predicted monotherapy failure, which may indicate good adjuvant therapy candidate.

## Graphical abstract

Extended robot‐assisted laparoscopic prostatectomy and extended pelvic lymph node dissection without adjuvant therapy is safe and effective for some patients with very‐high‐risk prostate cancer. The clinical stage and node positivity status predicted monotherapy failure, which may indicate good adjuvant therapy candidates.

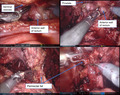

## 1 INTRODUCTION

The National Comprehensive Cancer Network (NCCN) guidelines classify prostate cancer (PCa) as “very high‐risk” if it includes at least one of the following: clinical stage cT3b‐T4, primary Gleason pattern 5, >4 cores with grade group 4 or 5, or more than one of the NCCN high‐risk characteristics.^1^ Patients with very‐high‐risk prostate cancer (VHRPCa) have significantly worse pathological outcomes than their high‐risk counterparts, such as earlier biochemical recurrences (BCRs) after radical prostatectomy (RP) and a higher mortality rate.^2^ The European Association of Urology and NCCN guidelines recommend RP with extended pelvic lymph node (LN) dissection (ePLND) as part of a multimodal approach in selected patients.^3^

Robot‐assisted laparoscopic prostatectomy (RALP) is the most common surgical procedure in men with localized PCa.^4^ Srougi et al., performed a systematic review and meta‐analysis and found that patients with high‐risk PCa treated with RALP have a lower risk of positive surgical margins (PSMs) and earlier BCR compared with those treated with open RP.^5^ However, there is still limited evidence to support the oncological efficacy of RALP in patients with VHRPCa.

RALP has several advantages, such as tissue magnification, recognition, and tridimensional vision, which enable very fine excision of tissue planes to maximize oncological control. Chao et al. analyzed the pathological features of a prostatectomy specimen from a patient with a locally advanced PCa and reported that the median extracapsular extension distance was 2.4 mm (range 0.05–7.0 mm).^6^ Therefore, wide resection of the surrounding prostate is required in patients with locally advanced PCa. Recently, a revised technique called extended RALP (eRALP) was reported and determined to be a safe and effective option for patients with locally advanced PCa.^7^, ^8^ However, these reports included patients that received neoadjuvant or adjuvant therapies. Therefore, it remains unknown whether eRALP as a monotherapy can improve the outcomes of patients with VHRPCa. This study analyzed a VHRPCa cohort treated using eRALP with ePLND without neoadjuvant or adjuvant therapy to assess the feasibility and efficacy of eRALP as monotherapy for VHRPCa.

## 2 MATERIALS AND METHODS

### 2.1 Patient population

Medical records were retrospectively reviewed between June 2016 and April 2020, and 84 patients who were classified as very‐high‐risk according to the NCCN guidelines were identified. Staging evaluations included computed tomography (CT) and magnetic resonance imaging (MRI), and a bone scan. Seventy‐six patients who were treated with eRALP‐ePLND without neoadjuvant or adjuvant therapy were included in the analysis. Eight patients who received neoadjuvant and adjuvant therapies were excluded. This observational study was approved by the Institutional Review Board (No: 1703014).

Preoperative and pathological data were collected from all patients and included age, prostate‐specific antigen (PSA) level at diagnosis, biopsy Gleason score (bGS), number of positive biopsy cores, clinical stage (cT), pathological stage (pT), pathological Gleason score (pGS), PSMs, number of nodes removed, and positive node (pN1) status. Perioperative outcomes included the operative time, blood loss, and 30‐day postoperative complications (using the Clavien–Dindo classification).

### 2.2 Surgical technique

**FIGURE 1:**
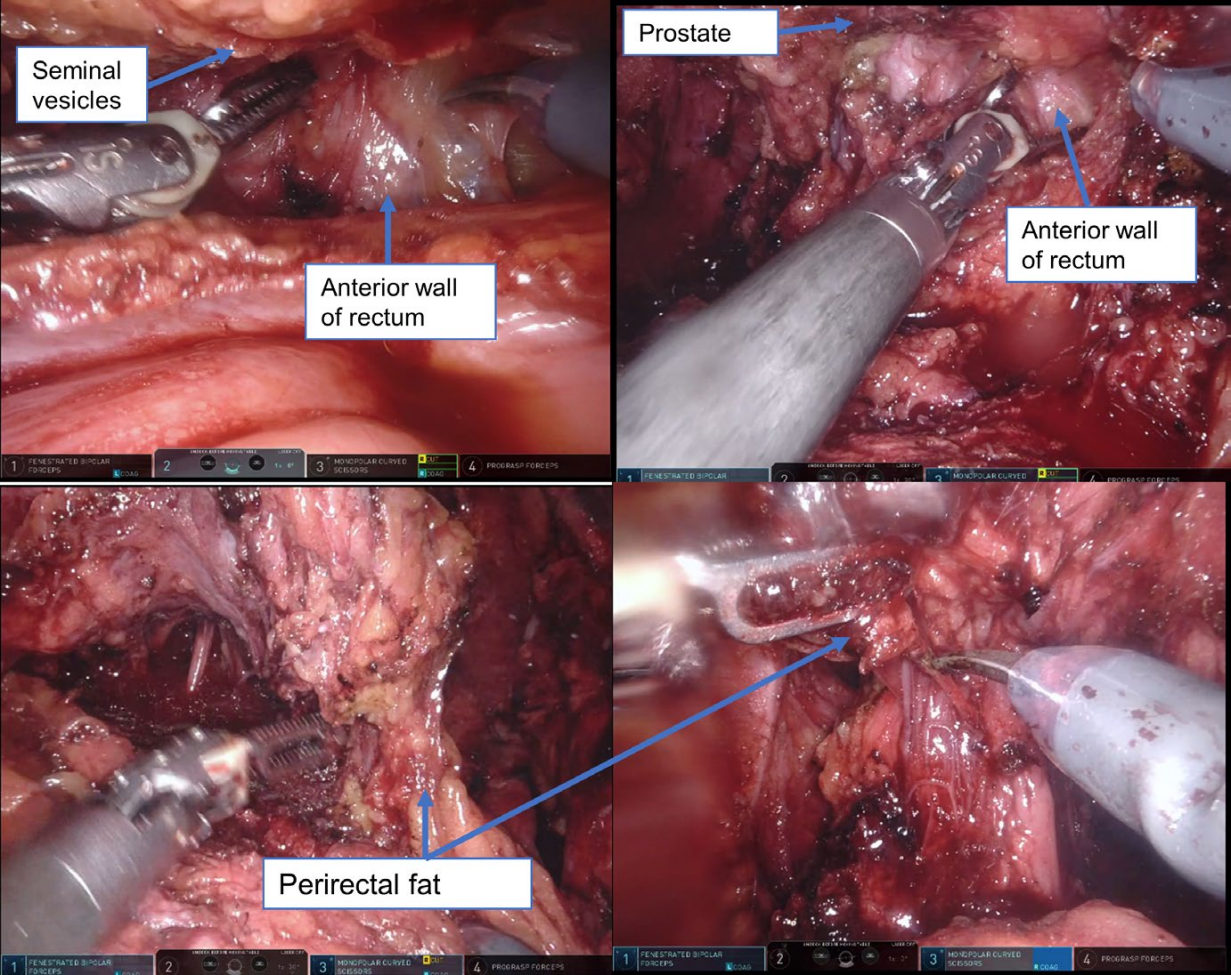
Extended robot‐assisted laparoscopic prostatectomy was performed using an extrafascial approach, where the dissection was carried forward to the anterior face of the rectum, pushing the perirectal fat and the Denonvilliers’ fascia upward with the specimen

Six surgeons performed the surgery under the supervision of one expert (T.S.). All procedures were performed using a DaVinci Si or Xi system (Intuitive Surgical, Sunnyvale, CA, USA) through a six‐port transperitoneal approach. eRALP was performed using an extrafascial approach, where the dissection was carried forward to the anterior face of the rectum, pushing the perirectal fat and the Denonvilliers’ fascia upwards with the specimen.^7^ This method can completely remove perirectal fat along with the prostate (Figure 1). ePLND included nodes above the external iliac axis, those in the obturator fossa, and around the internal iliac artery up to the ureter.^9^

### 2.3 Follow‐up

PSA measurements were monitored every 3 months during the first year and semiannually thereafter. BCR was defined as two consecutive PSA values of ≥0.2 ng/ml. Treatment failure was defined as when the PSA level did not decrease to <0.1 ng/ml postoperatively; in these instances, the BCR date was defined as the surgery date. sRT was defined as local radiation to the prostate and seminal vesicle bed when PSA elevated above 0.2 ng/ml or treatment failure. Castration‐resistant prostate cancer (CRPC) was defined as two consecutive increases in PSA levels despite castration testosterone levels (serum testosterone level, 0.50 ng/ml) and a baseline PSA level of ≥2 ng/ml.^10^ Clinical recurrence was defined as positive imaging during the follow‐up period after BCR onset. Bone scan and CT were performed in patients with BCR after RALP who had a high PSA (>1.0 ng/ml) or in patients with symptoms of bone disease. Clinical recurrence included both local and distant metastases. Urinary continence (UC) recovery was defined as using zero or one safety pad at the last follow‐up.^8^

### 2.4 Statistical analyses

Kaplan–Meier method and log‐rank test were used to assess the time to BCR and CRPC. Logistic regression analysis was used to determine preoperative risk factors in treatment failure. Cox proportional‐hazards model (with hazard ratio [HR]) was used to determine BCR risk factors. Multivariable Cox regression analyses were used to evaluate BCR predictors in the entire cohort. The covariates were PSA level, pathologic T stage, pathological primary Gleason grade 5, LN invasion, and PSMs. All *p*‐values were two‐sided, and statistical significance was defined as *p* < 0.05. Statistical analyses were performed using SPSS version 26.0 (IBM).

## 3 RESULTS

### 3.1 Patient characteristics

Preoperative characteristics are presented in Table 1. The median age was 70 years (interquartile range [IQR]: 67–75 years), and the median preoperative PSA was 20.0 (IQR: 7.1–38.9) ng/ml. In total, 92.1% of patients had a bGS of ≥8, and 36.8% had primary Gleason pattern 5 at biopsy. There were 32 patients (42.1%) in clinical stage ≤cT2, 33 (43.4%) in cT3a, 11 (14.5%) in cT3b, and 0 (0%) in cT4.

**TABLE 1:** Patient characteristics

	*N* = 76
Age (yr)/median (IQR)	70 (67–75)
PSA (ng/ml) median (IQR)	20.00 (7.10–38.88)
Biopsy Gleason score	
6 (%)	1 (1.3)
7 (%)	5 (6.6)
8–10 (%)	70 (92.1)
Primary Gleason grade 5 (%)	28 (36.8)
Number of biopsy cores median (IQR)	5 (4–7)
Clinical stage	
≤cT2	32 (42.1)
cT3a	33 (43.4)
cT3b	11 (14.5)
cT4	0 (0)
Very‐high‐risk factor	
Primary Gleason grade 5 (%)	28 (36.8)
>4 cores of GS 8–10 (%)	25 (32.9)
cT3b‐4 (%)	11 (14.5)
≥2 factors of NCCN high risk	29 (38.2)

IQR, interquartile range.

### 3.2 Perioperative outcomes

Perioperative parameters are presented in Table 2. The median operative time was 246 (IQR: 226–279) min, and the median blood loss was 50 (IQR: 0–100) ml. Twenty‐one patients (27.6%) experienced postoperative complications; nine patients were Clavien–Dindo grade I (11.8%), seven were grade II (9.2%), one was grade IIIa (1.3%), and four were grade IIIb (5.3%). Among grade III cases, two patients had a wound hernia (treated with surgical repair), one had urethral stenosis (treated with an internal urethrotomy), one had a rectal injury (treated with a temporary colostomy), and one had a pelvic lymphocele infection (treated with percutaneous drainage). There was no perioperative mortality.

**TABLE 2:** Perioperative outcomes of prostate cancer patients with very high‐risk prostate cancer treated with extended robot‐assisted laparoscopic prostatectomy

	*N* = 76
Operative time (min) median (IQR)	246 (226–279)
mean (±SD)	251(±44)
Blood loss (ml) median (IQR)	50 (0–100)
mean (±SD)	70(±92)
Postoperative complications (%)	22 (28.9%)
Clavien–Dindo classification (%)	
Ⅰ	9 (11.8%)
Ⅱ	7 (9.2%)
Ⅲa	1 (1.3%)
Ⅲb	4 (5.3%)
Ⅳ	0 (0%)
Ⅴ	0 (0%)
Postoperative complications	
Ⅲa Urethral stenosis	1
Ⅲb Wound hernia	2
Rectal injury	1
Infection of lymphocele	1

### 3.3 Pathological outcomes

**FIGURE 2:**
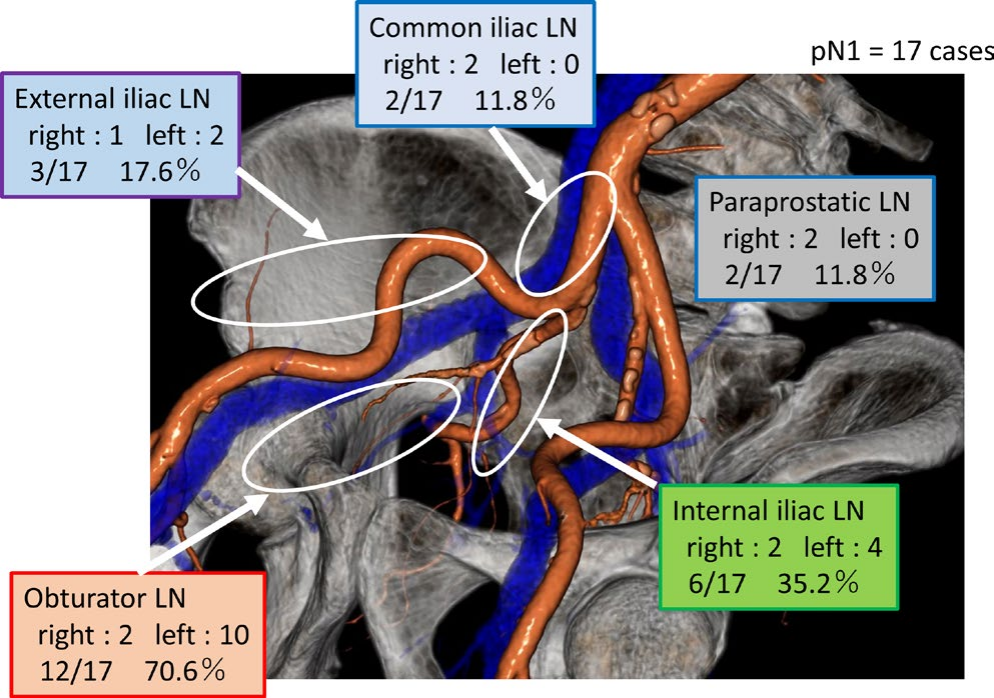
Distribution of node‐positive patients (pN1) undergoing extended pelvic lymph node dissection per region

Pathological outcomes are presented in Table 3. Histopathological evaluation classified 20 specimens (26.3%) as ≤pT2, 32 (42.1%) as pT3a, and 24 (31.6%) as pT3b. Thirty‐six patients with VHRPCa (47.4%) had specimen‐confined disease (i.e., negative margins and negative LNs). The PSM rate was 43.4% (*n* = 33), and the specific PSM locations were the apical side of prostate (13, 16.9%), rectal side (3, 3.9%), lateral side prostate (6, 7.8%), anterior side prostate (1, 1.3%), bladder neck (16, 20.8%), and seminal vesicle (1, 1.3%). The median number of removed nodes was 20. pN1 was found in 17 patients (22.4%), and the median number of positive nodes was two. The most frequent site of LN metastasis was the obturator LN in pN1 patients (12, 70.6%), followed by the internal iliac LNs (6, 35.2%), external iliac LNs (3, 17.6%), and common iliac (2, 11.8%) and paraprostatic LNs (2, 11.8%) (Figure 2).

**TABLE 3:** Pathological outcomes

	*N* = 76
Pathological stage (%)	
≤pT2	20 (26.3)
pT3a	32 (42.1)
pT3b	24 (31.6)
pT4	0 (0)
Pathological Gleason score (%)	
6	0 (0)
7	6 (7.9)
8–10	70 (92.1)
Positive surgical margins (%)	33 (43.4)
pN1	17 (22.4)
Number of nodes removed/median (IQR)	20 (15–29)
Number of positive nodes/median (IQR)	2 (1–3)
Specimen confined disease (%)	36 (47.4)

IQR, interquartile range.

### 3.4 Oncological and functional outcomes

**FIGURE 3:**
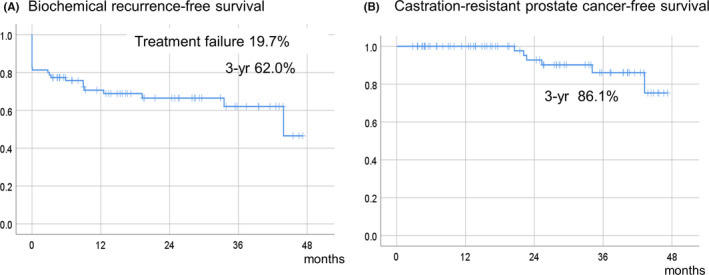
Kaplan–Meier analyses assessing the time to (a) biochemical recurrence and (b) castration‐resistant prostate cancer in patients with very‐high‐risk prostate cancer treated with extended robot‐assisted laparoscopic prostatectomy and extended pelvic lymph node dissection (median follow‐up, 25.2 months)

At 25.2 months (median follow‐up), 15 patients had treatment failure (19.7%), 26 had BCR (34.2%), and 5 had radiographic recurrence (6.6%). The radiographic recurrence locations were bone (one case), LN outside of the pelvic LN dissection (two cases), and the lung (one case). None of the patients received adjuvant therapy. Of the 26 patients with PSA recurrence, 17 underwent salvage androgen deprivation therapy (ADT), 2 sRT, 3 sRT followed by ADT, and four did not undergo salvage treatment. Of the 17 patients with pN1, 9 patients maintained without PSA recurrence and did not undergo salvage treatment. Eight patients had PSA recurrence, seven patients underwent salvage ADT and one underwent sRT. Postoperatively, the 3‐year, BCR‐free survival was 62.0%, CRPC‐free survival was 86.1% (Figure 3), and overall survival (OS) was 100% (data not shown). Multivariable logistic regression analysis showed that clinical stage of cT3b‐T4 was a preoperative independent predictor of treatment failure (HR 5.45, 95% confidence interval [CI] 1.17–25.43, *p* = 0.031) (Table 4). The Cox proportional‐hazards model showed that pN1 was an independent predictor of BCR among preoperative and pathological factors (HR 2.44, 95% CI 1.06–5.64, *p* = 0.037) (Table 5). Among 55 patients who had complete postoperative UC data, 47 patients (85.5%) recovered from their UC within 12 months. (Supplementary Figure S1).

**TABLE 4:** Univariate and multivariable logistic regression analyses assessing postoperative predictors of treatment failure in prostate cancer patients with very‐high‐risk prostate cancer treated with extended robot‐assisted laparoscopic prostatectomy

Baseline characteristics	Univariate analysis HR 95% CI *p*‐value			Multivariable analysis HR 95% CI *p*‐value		
Preoperative prostate‐specific antigen (ng/ml)	1.02	0.98–1.03	0.11	1.00	0.98–1.03	0.74
cT stage T3b‐T4 (vs≦T3a)	7.47	1.88–29.68	0.004	5.45	1.17–25.43	0.031
>4 cores of GS 8–10	1.47	0.46–4.73	0.52	1.33	0.37–4.78	0.67
Primary Gleason grade 5	0.36	0.092–1.41	0.14	0.53	0.12–2.33	0.40

**TABLE 5:** Univariate and multivariable Cox regression analyses assessing postoperative predictors of biochemical recurrence in patients with prostate cancer with very‐high‐risk prostate cancer treated with extended robot‐assisted laparoscopic prostatectomy

Baseline characteristics	Univariate analysis HR 95% CI *p*‐value			Multivariable analysis HR 95% CI *p*‐value		
Preoperative prostate‐specific antigen (ng/ml)	2.59	1.13–5.90	0.025	1.01	1.00–1.02	0.21
pT stage T3b‐T4 (vs≦T3a)	1.93	0.87–4.26	0.106	1.43	0.59–3.49	0.43
Prostatectomy Primary Gleason grade 5	1.27	0.56–2.89	0.561	1.53	0.65–3.56	0.33
Resection margin positive	2.16	0.97–4.79	0.062	2.13	0.96–4.69	0.062
pN1 (vs. pN0)	2.75	1.18–6.37	0.019	2.44	1.06–5.64	0.037

## 4 DISCUSSION

This study aimed to assess the feasibility and efficacy of eRALP‐ePLND monotherapy for VHRPCa and to identify patients who do not require multimodal approaches. The pathological results showed that approximately 50% of men who underwent only eRALP had specimen‐confined disease at the final pathological evaluation, despite having a VHRPCa diagnosis. Moreover, our results show that ePLND could achieve adequate nodal staging, as a median of 20 nodes were removed and the LN invasion rate was approximately 20%. The number of LN dissections was comparable with that of another study.^11^ Regarding oncological outcomes, approximately two‐thirds of patients were BCR‐free at the 3‐year follow‐up, and the CRPC‐free survival rates exceeded 85%. Moreover, perioperative outcomes were feasible, as only 6.6% of patients experienced Clavien‐Dindo Grade IIIa or IIIb complications within 30 days of surgery. Finally, more than 80% of patients had continence recovery within 12 months postoperatively. We found that eRALP‐ePLND was safe and had acceptable UC‐recovery rates, and that eRALP monotherapy‐controlled cancer progression in more than half of the patients with VHRPCa. Our results indicated that patients with specimen‐confined disease treated with eRALP‐ePLND could avoid additional treatment.

In patients with PCa who have PSA levels of >20 ng/ml, Gleason scores between 8 and 10, or clinical stages T3 or higher constitute a high‐risk PCa group, as recognized by major international guidelines.^1^, ^3^ Several studies reported that patients with high‐risk PCa have a heterogeneous natural history after RP.^12^, ^13^, ^14^ Saudi et al., used the Johns Hopkins RP database in 2014 to derive a binary definition of very‐high‐risk localized PCa. This definition was adopted by the NCCN to stratify patients into very‐high‐risk (VHR) and high‐risk (HiR) groups.^15^ They demonstrated that patients with VHRPCa presented with pN1 more frequently (VHR: 24.6%; HiR: 10.6%; *p *< 0.001) and had worse 5‐year biochemical relapse‐free survival (VHR: 27.5%; HiR: 50.3%, *p* < 0.001), 5‐year metastasis‐free survival (VHS: 74.9%; HiR: 91.4%, *p* < 0.001), and 5‐year OS (VHS: 85.8%; HiR: 95.1%, *p* < 0.001) than high‐risk patients. Pompe et al. also investigated patients in a European cohort and showed that only 20.6% of VHR cases had organ‐confined, LN‐negative PCa. Patients with VHRPCa also had worse pathological tumor features and a higher risk of adverse cancer outcomes compared with those with HR disease.^2^ Therefore, VHRPCa patients generally require additional therapies, such as ADT and RT. Recently, the results of multimodal treatment with adjuvant radio‐chemo‐hormone therapy have also been reported.^16^ The NCCN guidelines indicate RALP as one of the multimodal therapies in patients with VHRPCa who are expected to have a prognosis of 5 years or longer or symptomatic.

Some groups also reported that revised techniques, such as super‐extended RALP should be used to achieve specimen‐confined resection and to reduce the risk of recurrence and mortality.^7^, ^8^ Gandaglia et al. used data from a multi‐institutional database to demonstrate that extrafascial RALP was safe and oncologically effective in patients with PCa with locally advanced disease. At the 3‐year follow‐up, the BCR‐ and clinical recurrence‐free survival rates were 63.3% and 95.8%, respectively.^7^ Mazzone et al. reported that extrafascial RALP can be used to treat patients with posterior iT3a or iT3b lesions on preoperative MRI and was associated with good morbidity. At the 2‐year follow‐up, the BCR‐free and additional treatment‐free survival rates were 55% and 66%, respectively.^8^ A review of VHRPCa or locally advanced PCa studies showed that the PSM rate in extrafascial RALP ranged from 20.0% to 32.3%. Compared with reports of standard RP or standard RALP surgeries in VHRPCa or locally advanced PCa patients, extrafascial RALP had a relatively low proportion of PSMs and relatively good BCR‐free survival. The perioperative complication rate for extrafascial RALP was between 12.7% and 29%, and this was slightly higher than standard RALP (between 8.3% and 15.0%). However, the rate of high‐grade Clavien‐Dindo complications did not differ between standard surgery and extrafascial RALP (Table 6).^2^, ^7^, ^8^, ^15^, ^17^, ^18^, ^19^, ^20^ Most importantly, these reports include those patients with neoadjuvant and adjuvant therapies and are not the outcomes of eRALP alone. In our study, we presented patient outcomes using eRALP monotherapy.

**TABLE 6:** Results of series evaluating oncological outcomes of radical prostatectomy in the patients with very‐high‐risk prostate cancer or locally advanced prostate cancer

Author	*N*	Risk	Type of operation	Operative time (min)	RM1 (%)	pN1 (%)	BRFS rate (%)	Complications all grade
Pompe RS^2^	1369	VHRPCa	Standard RP	na	43.0	40.0	5 yr 43.1	na
Sundi D^15^	114	VHRPCa	Standard RP	na	26.3	24.6	5 yr 27.5	na
Ham WS^17^	121	≥cT3	sRALP	214	48.8	24.0	Na	8.3
Casey JT^18^	35	≥pT3	eRALP	271	20.0	19.0	Na	29.0
Vora AA^19^	140	≥pT3	sRALP	na	47.1	pNx	1 yr 50.0	na
Koo KC^20^	53	≥cT3b or cN1	sRALP	200	60.0	25	2 yr 55.0	15.0%
Gandaglia G^7^	94	VHRPCa	eRALP	233	32.3	37.2	3 yr 63.3	12.7%
Mazzone E^8^	89	cT3a‐b	eRALP	204	27.0	na	2 yr 55.0	21.4%
Present study	76	VHRPCa	eRALP	246	43.4	22.4	3 yr 62.0	28.9%

A primary benefit of radical surgery in patients with VHRPCa is the evaluation of surgical specimens. Accurate staging of progressive disease is important when making decisions about adjuvant therapy, and there is often a discrepancy between diagnostic imaging and pathological diagnosis^21^, ^22^ eRALP‐ePLND is the only treatment that enables clinical restaging with pathological findings. Therefore, this surgery may prevent over‐diagnosed patients from being unnecessarily exposed to the adverse effects inherent to adjuvant therapy. At the 3‐year follow‐up, almost 60% of patients with VHRPCa who were treated with eRALP maintained BCR‐free survival by surgery alone, and approximately 20% of patients had treatment failure, even with wide resection. Multivariable logistic regression analyses showed that stages cT3b‐T4 were preoperative independent predictors of treatment failure and pN1 was an independent predictor of BCR among other preoperative and pathological factors. Assessing these factors may help identify appropriate candidates for eRALP with neoadjuvant or adjuvant therapy.

There is increasing evidence that postoperatively combining RT and ADT throughout the pelvis improves the prognosis of pN1 disease. Abdollah et al. demonstrated that the adjuvant RT (aRT) survival benefits for pN1 patients with PCa were highly influenced by tumor characteristics. Men with low‐volume nodal disease (<3 LNs), International Society of Urological Pathology cancer grades 2–5, pathological stages pT3‐4, PSMs, or men with 3–4 positive nodes were more likely to benefit from RT after surgery, whereas the other subgroups were not.^23^ These results were confirmed by a comparative analysis of three current management strategies, including observation, ADT, and ADT +external beam radiation therapy (EBRT), using the United States National Cancer Database study that included 8074 pN1 patients. Immediate adjuvant therapies did not confer a significant OS benefit in men without adverse pathological features.^24^ Recently, Tilki et al. compared aRT therapy with initial observations and subsequent salvage RT (sRT) therapy in BCR cases when patients with PCa were LN‐positive at the time of RP. They showed that early sRT (pre‐RT PSA level ≤0.5 ng/ml) in patients with pN1 disease was significantly associated with decreased risk of metastasis compared with patients with late sRT (pre‐RT PSA level >0.5 ng/ml).^25^ Accurate pathological diagnosis along with eRALP‐ePLND and careful observation of postoperative PSA changes may allow better adjuvant therapy selection for pN1 PCa patients. The eRALP‐ePLND allows correct staging in up to 94% of patients with node‐positive disease and removes all metastatic nodes in approximately 75% of cases.^26^ The comparative oncological effectiveness of RP as part of a multimodal treatment strategy versus upfront EBRT with ADT for locally advanced PCa remains unknown. However, a prospective phase‐III randomized clinical trial (SPCG‐15) comparing RP (with or without adjuvant or salvage EBRT) against primary EBRT and ADT among patients with locally advanced (T3) disease is currently recruiting participants (https://clinicaltrials.gov/ct2/show/NCT02102477).

Our analysis has some limitations. We analyzed retrospective data, and the cohort had a relatively short follow‐up period. Therefore, the generalizability of the results is partially limited. Owing to the small number of events, the multivariable Cox model suffers from consistent overfitting, also limiting the generalization of the findings. Therefore, it should be validated in a larger cohort patient with VHRPCa. The timing and type of additional treatments after recurrence was determined by the individual's physician, and the variability may affect the reported results. Furthermore, it is still unclear whether the oncological effects are superior in patients with neoadjuvant/adjuvant therapy or patients with eRALP alone. Further research is needed to confirm the effectiveness of eRARP‐ePLND in a long‐term follow‐up study with patients with VHRPCa.

## 5 CONCLUSIONS

We analyzed a cohort of patients with VHRPCa treated with eRALP‐ePLND monotherapy. This approach was safe and produced acceptable UC recovery rates. Furthermore, this approach accomplished good oncological control in patients without preoperative SVI or pathological LN metastases at intermediate‐term follow‐up. We found two perioperative predictive factors: clinical stage cT3b‐T4 indicated treatment failure and pN1 indicated BCR. Therefore, our results may contribute to informed decisions about neoadjuvant or adjuvant therapies in patients with VHRPCa. Further updates are needed to improve follow‐up time and patient numbers.

## ETHICS STATEMENT

This study was approved by the Institutional Review Board (No: 1703014) at Ehime University Hospital.

## CONFLICT OF INTEREST

The authors have no conflicts of interest in this work.

## AUTHOR CONTRIBUTIONS

Project development: N Miura, T Saika; Data collection: N Miura, K. Funaki, N Sugihara, T. Kakuta, K Koyama, Y Sawada, T Noda, K Nishimura, T Fukumoto, Y Miyauchi, T Kikugawa; Data analysis: N Miura, Y Miyauchi; Manuscript writing/editing: N Miura, T Saika.

## Supporting information

Fig S1

## Data Availability

The data that support the findings of this study are available from the corresponding author upon reasonable request.
