# Supplementary figures and images for: Extended robot‐assisted laparoscopic prostatectomy and extended pelvic lymph node dissection as a monotherapy in patients with very high‐risk prostate cancer Patients
Source: Cancer Med. 2021 Sep 25;10(22):7968–76. doi: 10.1002/cam4.4308 (PMC8607267; doi:10.1002/cam4.4308)

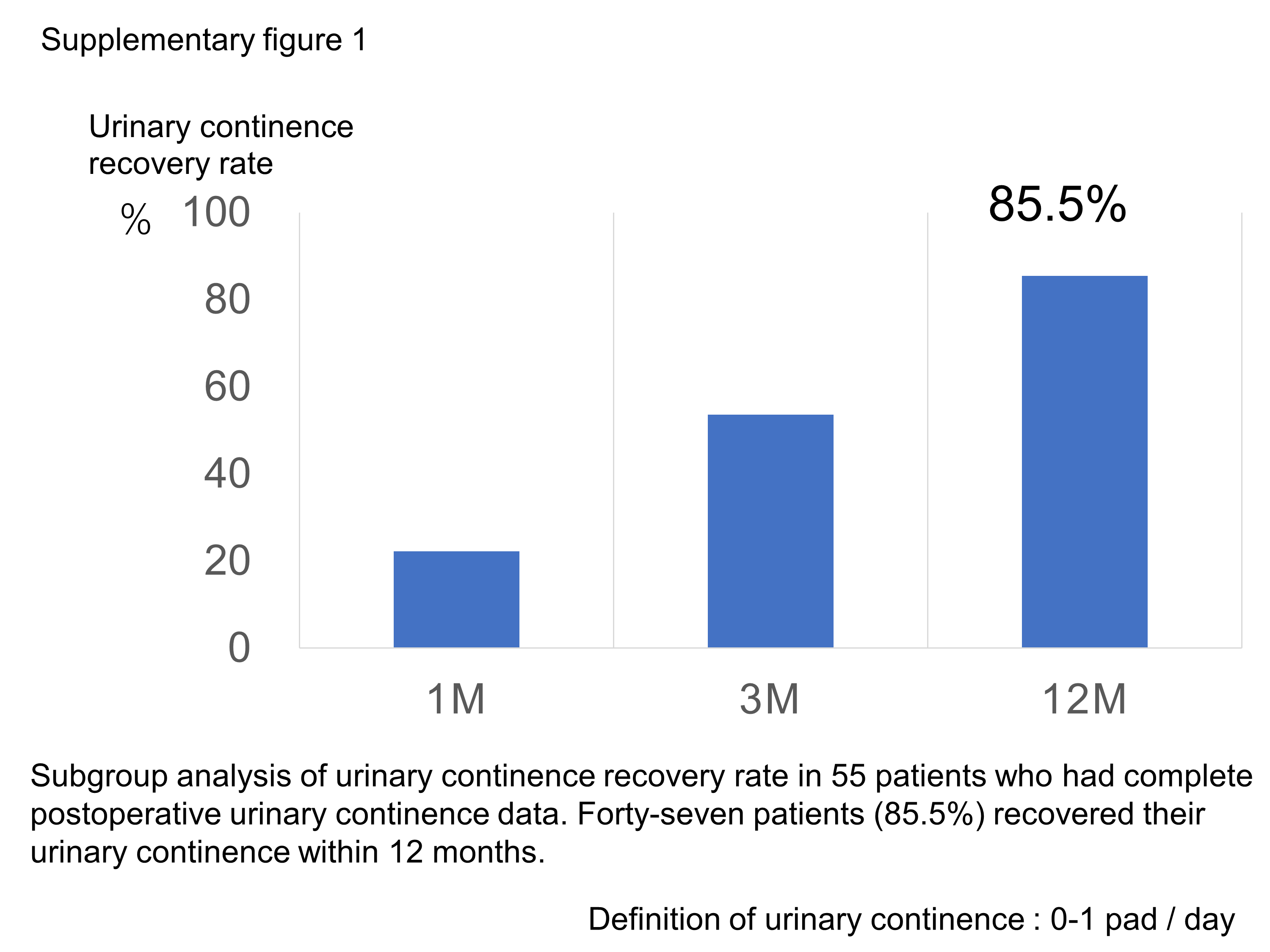

Supplement: Supplementary file 1 — Fig S1 [file CAM4-10-7968-s001.tif]
